# VHL status regulates transforming growth factor-β signaling pathways in renal cell carcinoma

**DOI:** 10.18632/oncotarget.24631

**Published:** 2018-03-27

**Authors:** Pramod Mallikarjuna, Raviprakash T. Sitaram, Maréne Landström, Börje Ljungberg

**Affiliations:** ^1^ Department of Medical Biosciences, Pathology, Umeå University, Umeå SE-90187, Sweden; ^2^ Department of Surgical and Perioperative Sciences, Urology and Andrology, Umeå University, Umeå SE-90187, Sweden

**Keywords:** ccRCC, non-ccRCC, ALK5, pVHL, TGF-β signaling

## Abstract

To evaluate the role of pVHL in the regulation of TGF-β signaling pathways in clear cell renal cell carcinoma (ccRCC) as well as in non-ccRCC; the expression of pVHL, and the TGF-β pathway components and their association with clinicopathological parameters and patient’s survival were explored. Tissue samples from 143 ccRCC and 58 non-ccRCC patients were examined by immunoblot. ccRCC cell lines were utilized for mechanistic *in-vitro* studies. Expression levels of pVHL were significantly lower in ccRCC compared with non-ccRCC. Non-ccRCC and ccRCC pVHL-High expressed similar levels of pVHL. Expression of the TGF-β type I receptor (ALK5) and intra-cellular domain were significantly higher in ccRCC compared with non-ccRCC. In non-ccRCC, expressions of ALK5-FL, ALK5-ICD, pSMAD2/3, and PAI-1 had no association with clinicopathological parameters and survival. In ccRCC pVHL-Low, ALK5-FL, ALK5-ICD, pSMAD2/3, and PAI-1 were significantly related with tumor stage, size, and survival. In ccRCC pVHL-High, the expression of PAI-1 was associated with stage and survival. *In-vitro* studies revealed that pVHL interacted with ALK5 to downregulate its expression through K48-linked poly-ubiquitination and proteasomal degradation, thus negatively controlling TGF-β induced cancer cell invasiveness. The pVHL status controls the ALK5 and can thereby regulate the TGF-β pathway, aggressiveness of tumors, and survival of the ccRCC and non-ccRCC patients.

## INTRODUCTION

Renal cell carcinoma (RCC) is a heterogeneous tumor caused by alterations in different genes. Based on various histological appearance and cytogenetic abnormality, RCC is classified into several subtypes [1, 2]. Main RCC types are clear cell RCC (ccRCC), papillary (pRCC), and chromophobe RCC (chRCC) [3]. Clear cell type is the most frequent RCC, accounting for 70-80% of RCCs, having a five years overall survival rate of about 55-60%. Papillary type represents 15-20% of RCC with five years survival rate about 70-90%, and chromophobe type counts for 6-11% of RCC with a five years survival rate of 80-95%. Both pRCC and chRCC revealed the hypovascular feature unlike ccRCC [4]. The differences in prognosis and tumor behavior of the different RCC types are due to the involvement of various genes and signaling pathways [5]. The pRCC is associated with germline mutations of *MET* proto-oncogene, and these mutations activate MET signaling to promote tumor and cell motility [6]. Recurrent genetic alterations found in chRCC are the loss of heterozygosity (LOH) at chromosomes 1, 2, 6, 10, 13, 17 and 21, and are associated with Brit Hogg Dube syndrome [6].

In ccRCC, common genetic aberrations are LOH, hypermethylation, or mutation or deletions in the 3p chromosome region [7]. Frequent aberrations of chromosome 3p region cause inactivation of von Hippel–Lindau (*VHL*) gene in ccRCC. In contrast, *VHL* is unaltered in pRCC and chRCC. The VHL protein (pVHL), encoded by the tumor suppressor gene *VHL*, serves as an adaptor protein like E3-ubiquitin ligase complex and targets HIF subunits for degradation by ubiquitination [8, 9]. In cases with inactive or absent pVHL, stabilization of Hypoxic α units (HIF-1 and HIF-2) occurs, leading to tumor forming and tumor promoting properties of ccRCC [10, 11]. The pVHL mediates both K48- and K63-linked poly-ubiquitination, which is involved in protein degradation, protein trafficking, and post-translational modification respectively [12]. Besides interaction with HIF-α, pVHL also associates with various proteins and mediates different biological processes [8]. In RCC, pVHL might negatively modulate transforming growth factor-β (TGF-β) pathways [13, 14].

TGF-β is a key regulatory cytokine controlling vital processes, such as immune responses, differentiation of cells, and tissue homeostasis. Initially, TGF-β acts as a tumor suppressor, but in the later stages of the tumor, TGF-β acts as a tumor promoter [15]. TGF-β signals through canonical and non-canonical modes [16–18]. In canonical signaling, TGF-β ligand binds to a constitutively active TGF-β type II receptor (TβRII); TBRII thereafter recruits and activates TGF-β type I receptor (TBRI or ALK5 or ALK5-Full Length; ALK5-FL) by phosphorylation in its serine/glycine-rich sequence called the GS domain. Activated TβRI then activates SMAD2 and SMAD3, which translocates to the nucleus together with SMAD4 to turn on target genes in a contextual manner [15]. In non-canonical signaling, TβRI is cleaved by proteolytic enzymes to liberate an intracellular domain (TβRI-ICD or ALK5-ICD) [19, 20]. Though the exact tumor promoting mechanism of ALK5-ICD needs to be further elucidated, studies have established the ability of ALK5-ICD to promote cancer and its localization in the nucleus of tumor cells [18–20]. The TGF-β signaling imparts its impact as a tumor promoter by activating tumor-promoting genes in the nucleus [20]. One of the well-known targets of TGF-β signaling is Plasminogen activator inhibitor type-1 (*PAI-1*) [21]. Increased expression of PAI-1 is seen in various forms of cancer [22–24], and it is also associated with poor prognosis in ccRCC [25, 26]. Recently, we investigated canonical and non-canonical components of TGF-β signaling pathway and demonstrated the poor survival imparted by TGF-β signaling and PAI-1, in patients with ccRCC [25].

In the present study, we investigate the role of TGF-β signaling pathways in non-ccRCC, having normal functioning pVHL. We also investigated the regulation of TGF-β signaling in ccRCC, based on the levels of pVHL expression. Further, to elucidate the mode of action of TGF-β and it’s abilities to induce tumor aggressiveness depending on the levels of pVHL expression, we utilized two ccRCC cell lines A498 (VHL^-/-^) and ACHN (VHL^+/+^) for *in-vitro* studies.

## RESULTS

### Expression patterns of pVHL in ccRCC and non-ccRCC

**Figure 1:**
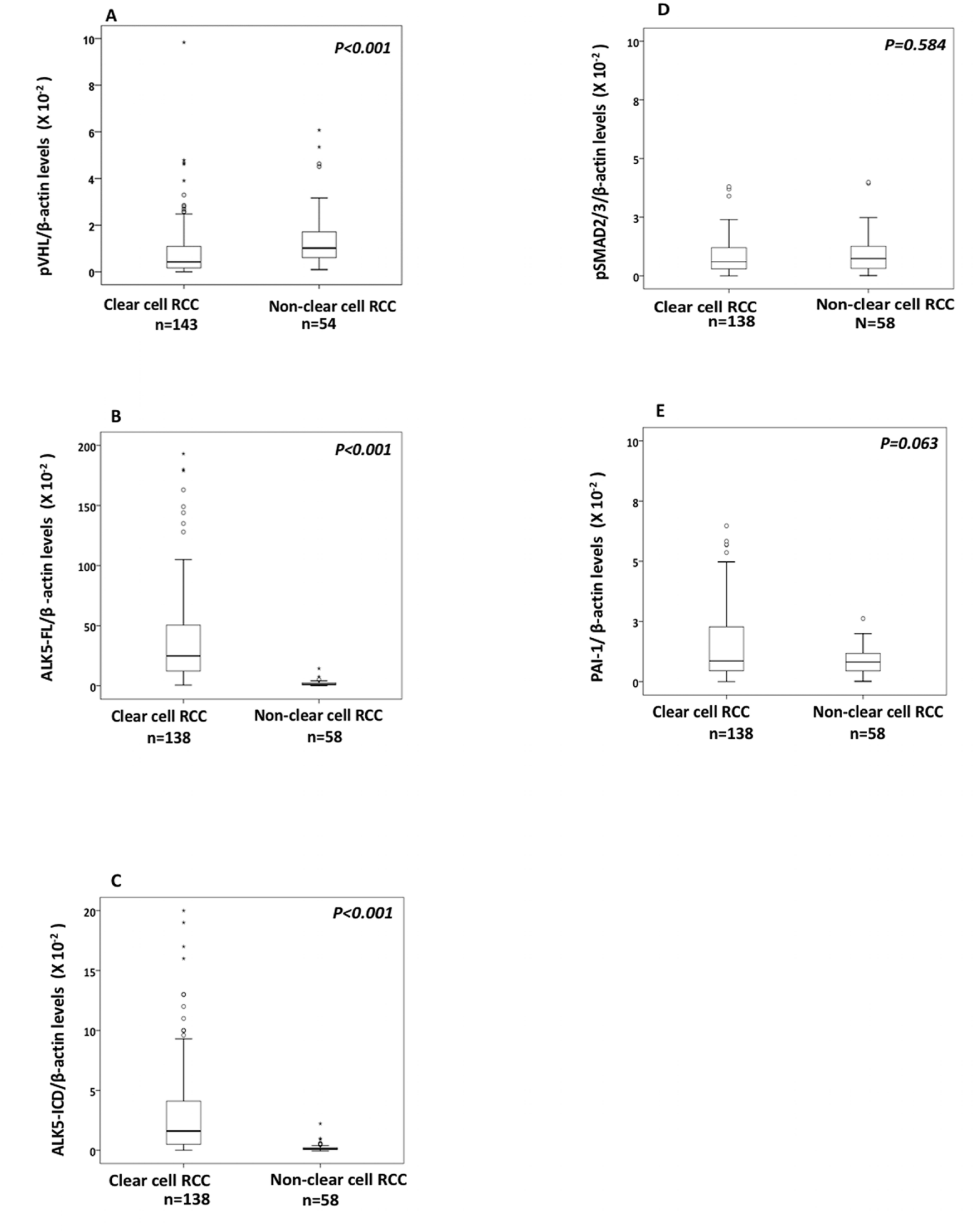
Box plot representation of expression of **(A)** pVHL, **(B)** ALK5-FL, **(C)** ALK5-ICD, **(D)** pSMAD2/3, **(E)** PAI-1 protein in the non-ccRCC compared with ccRCCs.

The pVHL levels were significantly lower in ccRCC (n=143) than its corresponding kidney cortex (n=35) (*P=0.012*), as well as non-ccRCC (n=54) (*P<0.001*, Figure 1A). For non-ccRCC (n= 54) there was no difference in pVHL levels (*P=0.663*) when compared with corresponding kidney cortex (n=20).

Based on the median value of pVHL expression, the ccRCCs (n=143) were divided into two subgroups: ccRCC pVHL-Low (n=71) and ccRCC pVHL-High (n=72). There were similar pVHL levels (*P=0.375*) expressed in ccRCC pVHL-High (n=72) and non-ccRCC (n=54), while ccRCC pVHL-Low had significantly lower pVHL levels than non-ccRCC (n=71) (*P< 0.001)*. Expression levels of pVHL showed no association with clinicopathological parameters either in ccRCC irrespective of pVHL levels, or in non-ccRCC (Table 1) (Supplementary Figure 1A and Supplementary Figure 1B).

**Table 1:** Relation of pVHL levels to categorized clinicopathological parameters in ccRCC and non-ccRCC

Parameter	ccRCC pVHL			ccRCC pVHL-Low			ccRCC pVHL-High			Non-ccRCC pVHL		
	n^a^	Mean Rank	*P*-Value^b^	n^a^	Mean Rank	*P*-Value^b^	n^a^	Mean Rank	*P*-Value^b^	n^a^	Mean Rank	*P*-Value^b^
**Grade**												
I (I+II)	72	74.25		36	36.64		36	40.86		32	29.23	
			0.513			0.791			0.077			0.328
II (III+IV)	71	69.72		35	35.34		36	32.14		22	24.98	
**TNM Stage**												
Early Stage (I+II)	79	70.03		40	33.48		39	36.51		38	29.86	
			0.527			0.242			0.995			0.090
Advanced Stage (III+IV)	64	74.44		31	39.26		33	36.48		16	21.91	
**Tumor size (mm)**												
<70	72	67.57		41	35.07		31	39.55		33	28.89	
			0.198			0.658			0.283			0.414
>70	71	76.49		30	37.27		41	34.20		21	25.31	

^a^ Number of patients.

^b^ Groups were compared using Mann-Whitney U test (significant at *P<0.05*).

### Expression pattern of ALK5-FL, ALK5–ICD, pSMAD2/3 and PAI-1 protein levels in ccRCC and non-ccRCC

The protein expression of ALK5-FL and ALK5-ICD were significantly higher (*P<0.001*) in ccRCC compared with non-ccRCC (Figure 1B and 1C), while there was no difference in the expression levels of pSMAD2/3 (*P=0. 584*) and PAI-1 (*P=0.063*) (Figure 1D and 1E).

### Expression of ALK5-FL, ALK5-ICD, pSMAD2/3, PAI-1 proteins and their relation with clinicopathological parameters

Protein levels of ALK5-FL, ALK5-ICD, pSMAD2/3, and PAI-1 in non-ccRCC did not associate with any of the clinicopathological parameters (Table 2).

**Table 2:** Relation of ALK5-FL, ALK5-ICD, pSMAD2/3 and PAI-1 protein levels to categorized clinicopathological parameters in non-ccRCC

Parameter	ALK5-FL			ALK5-ICD			pSMAD2/3			PAI-1		
	n^a^	Mean Rank	*P*-Value^b^	n^a^	Mean Rank	*P*-Value^b^	n^a^	Mean Rank	*P*-Value^b^	n^a^	Mean Rank	*P*-Value^b^
**Grade**												
I (I+II)	33	28.58		33	32.56		33	27.82		33	30.82	
			0.632			0.113			0.384			0.495
II (III+IV)	25	30.72		25	25.46		25	31.43		25	27.76	
**TNM Stage**												
Early Stage (I+II)	40	28.38		40	32.16		40	27.58		40	29.88	
			0.449			0.073			0.196			0.801
Advanced Stage (III+IV)	18	32		18	23.58		18	33.78		18	28.67	
**Tumor size (mm)**												
<70	35	28.74		35	29.21		35	28.23		35	30	
			0.674			0.874			0.479			0.781
>70	23	30.65		23	29.93		23	31.43		23	28.74	

^a^ Number of patients.

^b^ Groups were compared using Mann-Whitney U test (significant at *P<0.05*).

In ccRCC pVHL-Low, the expression of ALK5-FL, ALK5-ICD, and pSMAD2/3 were significantly associated with the tumor stage and tumor diameter. However, PAI-1 was not only associated with the tumor stage and diameter but also with the tumor grade (Table 3). In ccRCC pVHL-High, none of the TGF-β signaling components were associated with clinicopathological parameters except ALK5-ICD, which associated with the tumor stage, and PAI-1 correlated with the tumor grade and stage (Table 4).

**Table 3:** Relation of ALK5-FL, ALK5-ICD, pSMAD2/3 and PAI-1 protein levels to categorized clinicopathological parameters in ccRCC VHL-Low

Parameter	ALK5-FL			ALK5-ICD			pSMAD2/3			PAI-1		
	n^a^	Mean Rank	*P*-Value^b^	n^a^	Mean Rank	*P*-Value^b^	n^a^	Mean Rank	*P*-Value^b^	n^a^	Mean Rank	*P*-Value^b^
**Grade**												
I (I+II)	34	30.85		34	30.49		34	30.03		34	28	
			0.128			0.093			0.061			0.007
II (III+IV)	34	38.15		34	38.51		34	38.97		34	41	
**TNM Stage**												
Early Stage (I+II)	38	28.50		38	29.53		38	26.70		38	26.49	
			0.005			0.019			0.000			0.000
Advanced Stage (III+IV)	30	42.10		30	40.80		30	44.38		30	44.65	
**Tumor size (mm)**												
<70	39	27		39	29.05		39	28.12		39	27.33	
			0.000			0.008			0.002			0.001
>70	29	44.59		29	41.83		29	43.09		29	44.14	

^a^ Number of patients.

^b^ Groups were compared using Mann-Whitney U test (significant at *P<0.05*).

**Table 4:** Relation of ALK5-FL, ALK5-ICD, pSMAD2/3 and PAI-1 protein levels to categorized clinicopathological parameters in ccRCC VHL-High

Parameter	ALK5-FL			ALK5-ICD			pSMAD2/3			PAI-1		
	n^a^	Mean Rank	*P*-Value^b^	n^a^	Mean Rank	*P*-Value^b^	n^a^	Mean Rank	*P*-Value^b^	n^a^	Mean Rank	*P*-Value^b^
**Grade**												
I (I+II)	34	35.78		34	32.24		34	33.47		34	30.13	
			0.911			0.206			0.416			0.032
II (III+IV)	36	35.24		36	38.49		36	37.42		36	40.57	
**TNM Stage**												
Early Stage (I+II)	37	34.12		37	30.11		37	35.20		37	29.46	
			0.548			0.019			0.897			0.009
Advanced Stage (III+IV)	33	37.05		33	41.55		33	35.83		33	42.27	
**Tumor size (mm)**												
<70	29	38.47		29	37.62		29	36.03		29	36	
			0.305			0.463			0.853			0.863
>70	41	33.40		41	34		41	35.12		41	35.15	

^a^ Number of patients.

^b^ Groups were compared using Mann-Whitney U test (significant at *P<0.05*).

### Protein levels of pVHL, ALK5-FL, ALK5-ICD, pSMAD2/3, and PAI-1 and their relation with cancer-specific survival

**Figure 2:**
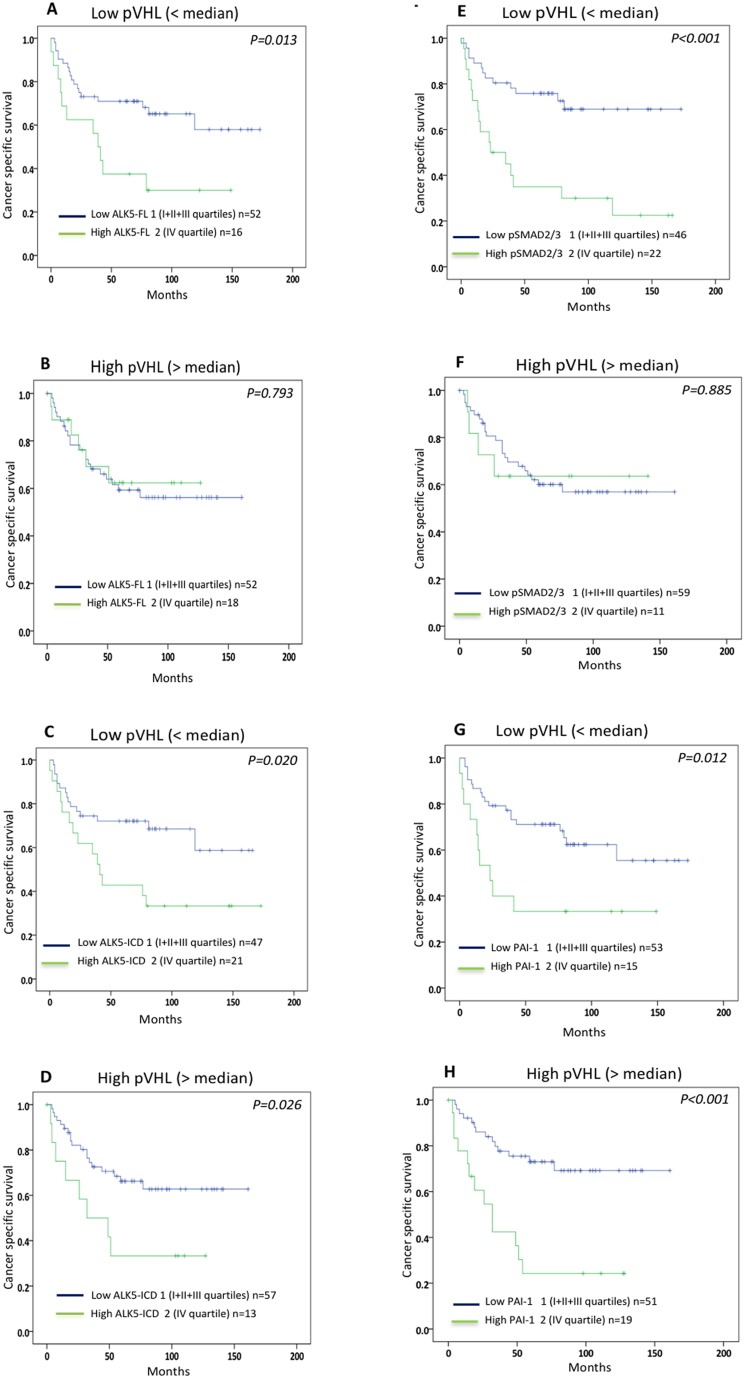
Kaplan-Meier plots showing cancer-specific survival curves of **(A, B)** ALK5-FL; **(C, D)** ALK5-ICD; **(E, F)** pSMAD2/3; (G, H) PAI-1 protein levels in ccRCC VHL- Low and ccRCC VHL-High.

Expression of pVHL did not associate with cancer-specific survival in patients with ccRCC or in patients with non-ccRCC. The expression levels of ALK5-FL, ALK5-ICD, pSMAD2/3 and PAI-1 protein showed no association with survival in the non-ccRCC patients. In contrast, expression levels of ALK5-FL, ALK5-ICD, pSMAD2/3 and PAI-1 proteins correlated with poor survival of ccRCC pVHL-Low patients. Notably, protein levels of ALK5-ICD and PAI-1 correlated with poor survival in ccRCC pVHL-High patients, while ALK5-FL and pSMAD2/3 showed no correlation with survival (Figure 2A-2H).

### VHL status regulated downstream signaling targets of TGF-β in ccRCC cell lines

**Figure 3:**
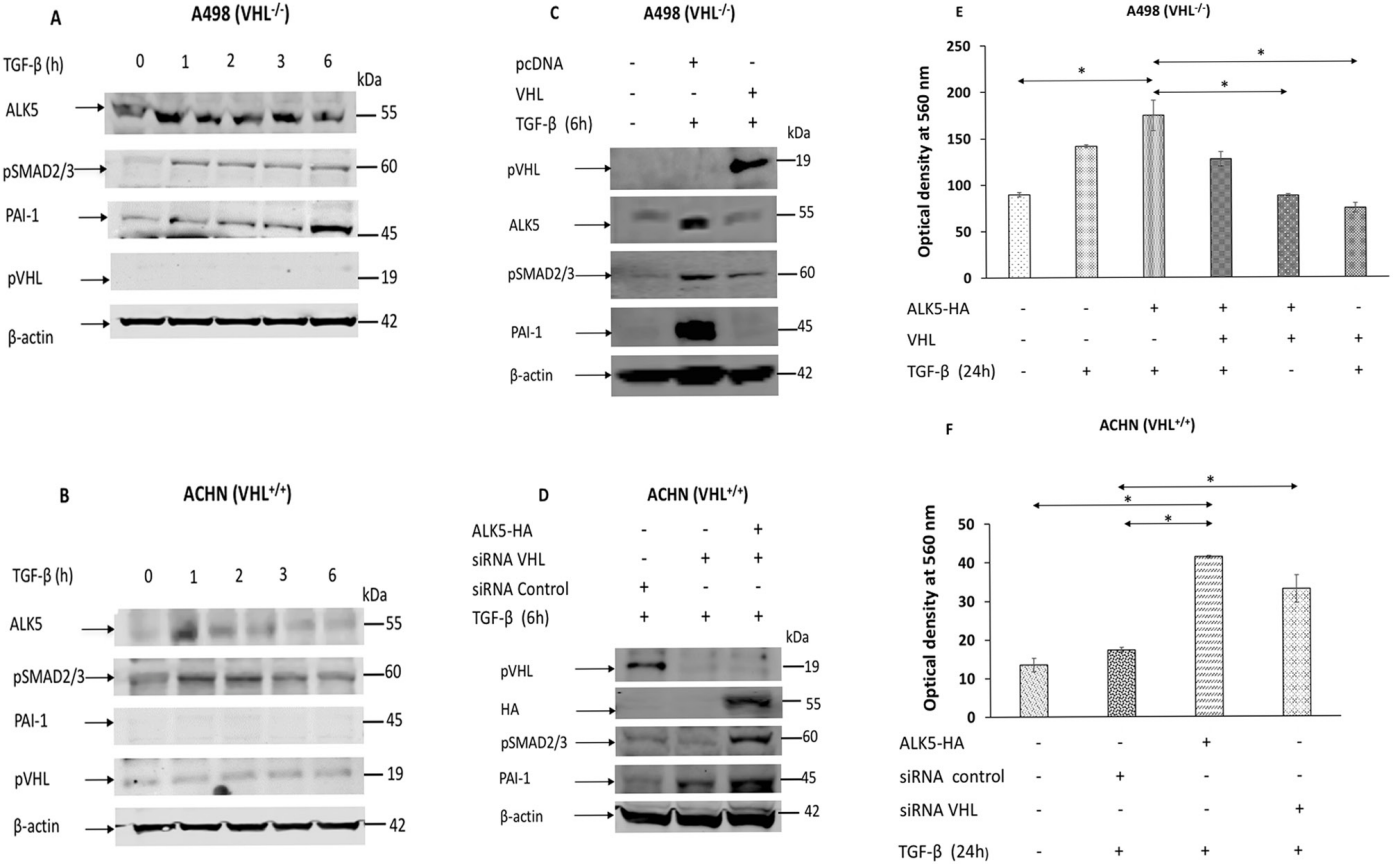
**(A)** Immunoblots showing protein expression of ALK5, pSMAD2/3, PAI-1, pVHL, and β-actin in A498 cells after treatment with TGF-β at given point of time; **(B)** Immunoblots showing protein expression of ALK5, pSMAD2/3, PAI-1, pVHL, and β-actin in ACHN cells after treatment with TGF-β at given point of time; **(C)** Immunoblots showing protein expression of pVHL, ALK5, pSMAD2/3, PAI-1, and β-actin after transfection of indicated vectors in A498 cells followed by TGF-β treatment for 6 hours; **(D)** Immunoblots showing protein expression of pVHL, HA, pSMAD2/3, PAI-1, and β-actin after transfection of indicated vectors in ACHN cells (48h) followed by TGF-β treatment for 6 hours; **(E)** Invasion assay showing the invasiveness induced by TGF-β in A498 cells after re-introduction of *VHL* (n=3 independent experiments ^*^P< 0. 05); **(F)** Invasion assay showing invasiveness by TGF-β in ACHN cells after *siRNA VHL* knockdown (n=3 independent experiments ^*^P< 0.05).

The A498 (VHL^-/-^) and ACHN (VHL^+/+^) cell lines were treated with TGF-β at indicated time points. In A498 cells, TGF-β treatment increased the protein level of PAI-1 in a time dependent manner, but not in ACHN cells (Figure 3A and 3B, n=3 independent experiments).

In A498 cells, treatment with TGF-β induced the expression of PAI-1 protein level, but the introduction of *VHL* along with TGF-β treatment, reduced the expression PAI-1 protein level as well as the TGF-β induced expression of endogenous ALK5 (Figure 3C, n=3 independent experiments). In ACHN cells, cells treated with vehicle control (siRNA control) showed no expression of the PAI-1 protein, but knockdown of *VHL* by siRNA showed expression of the PAI-1 protein. Further, knockdown of *VHL* by siRNA and overexpression of ALK5, followed by TGF-β treatment for 6 hours, enhanced the expression of PAI-1 protein level (Figure 3D, n=3 independent experiments).

Collectively, these results indicate that VHL status influences the efficiency of TGF-β to induce its downstream targets in ccRCC cell lines.

### VHL suppressed the invasion of ccRCC cell lines induced by TGF-β

In A498 cells, overexpression of *ALK5-HA* followed by treatment with TGF-β increased the invasiveness of cells. However, co-transfection of *VHL* and *ALK5-HA* followed by treatment with TGF-β, or overexpression of *VHL* alone, followed by treatment with TGF-β significantly reduced the invasiveness of cells when compared with cells transfected with *ALK5-HA* followed by treatment with TGF-β (Figure 3E, n=3 independent experiments). In the absence of TGF-β treatment, cells transfected with *VHL* showed reduced invasion when compared with cells transfected with pcDNA control of ALK5 (Supplementary Figure 2, n=3 independent experiments). These results showed that the pVHL negatively regulated the TGF-β induced invasiveness in A498 cells.

In ACHN cells, TGF-β treatment did not induce the invasiveness of cells transfected with siRNA control. However, overexpression of *ALK5-HA* and followed by TGF-β treatment significantly increased the invasiveness of cells when compared with untreated control cells, or cells transfected with siRNA control and treated with TGF-β. These results revealed that the presence of wild-type *VHL* reduced the invasiveness induced by TGF-β, while overexpression of ALK5 and treatment with TGF-β increased the invasiveness. Importantly, knockdown of *VHL* through siRNA followed by TGF-β stimulation, significantly increased the invasiveness of cell. This result indicated that knockdown of *VHL* alone is sufficient to sensitize ACHN cells to TGF-β stimulation (Figure 3F, n=3 independent experiments).

Collectively, these results showed that both the wild-type *VHL* and re-introduced *VHL*, will render cells unresponsive to invasiveness due to TGF-β treatment for 24 hours.

### pVHL associates with ALK5

**Figure 4:**
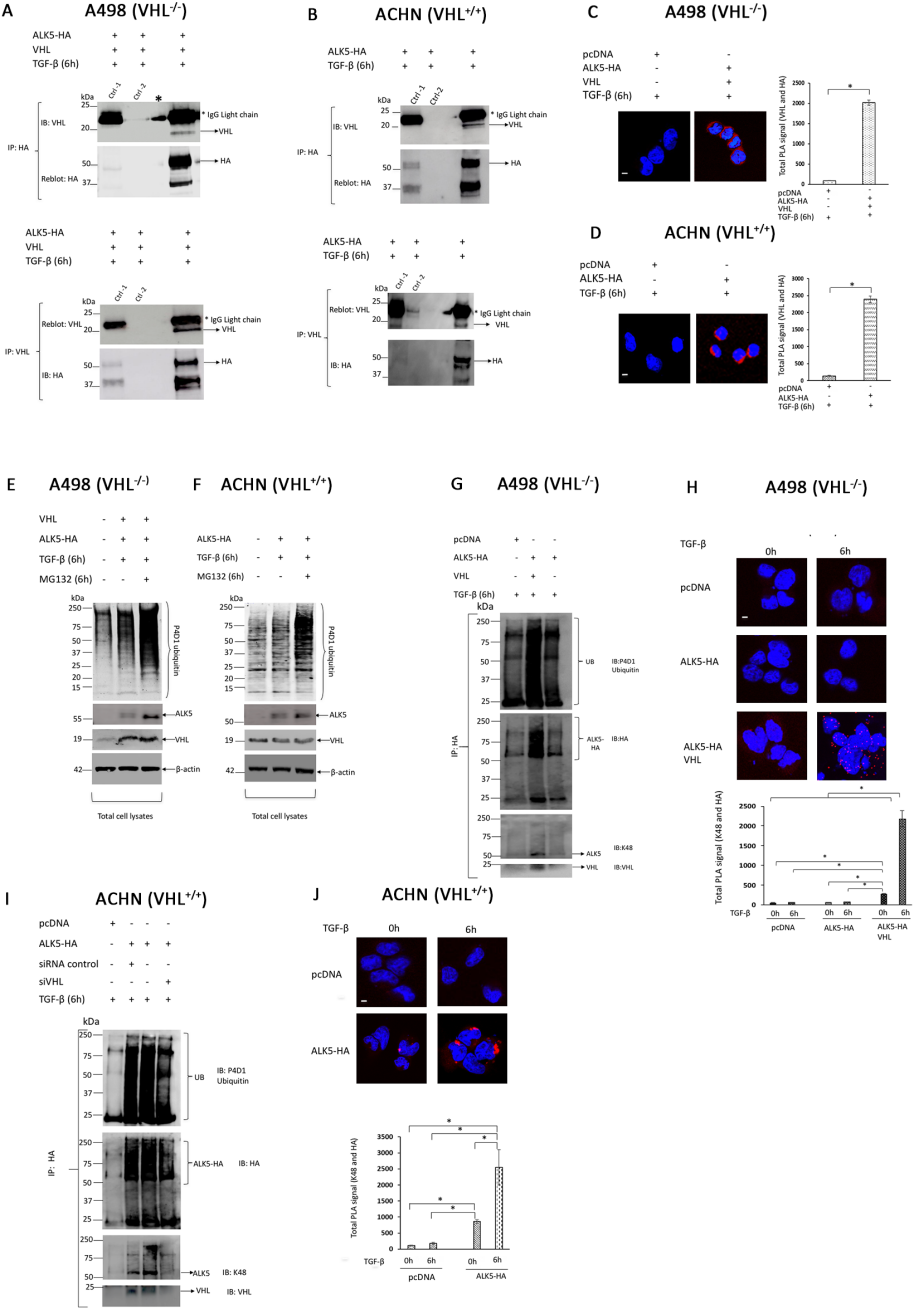
**(A)** Immunoblots showing IP with HA or VHL antibody: A498 cells were co-transfected with *ALK5-HA* and *VHL* vectors, followed by TGF-β treatment for 6h, and probed with HA or VHL antibody, respectively (^*^ indicates a spill, Ctrl-1 is isotype specific IgG plus beads, Ctrl -2 is beads only); **(B)** Immunoblots showing IP with HA or VHL antibody: ACHN cells were transfected with *ALK5-HA* vector, followed by TGF-β treatment for 6h, and probed with HA or VHL antibody, respectively (Ctrl-1 is isotype specific IgG plus beads, Ctrl -2 is beads only); **(C)** A498 cells were transiently co-transfected with *ALK5-HA* and *VHL* vectors or *pcDNA 3. 1(+)* empty vector, followed by TGF-β treatment for 6h, and pVHL-ALK5-HA complexes was visualized by staining cells after probing against VHL and HA (red) antibodies followed by PLA. Scale bar 20 μm (mean ± SD of three experiments, 100 cells were analyzed in each group); **(D)** ACHN cells were transfected with *ALK5-HA* or *pcDNA 3.1(+)* empty vector, followed by TGF-β treatment for 6 h, and pVHL-ALK5-HA complexes was visualized by staining cells after probing against VHL and HA (red) antibodies followed by PLA. Scale bar 20 μm (mean ± SD of three experiments, 100 cells were analyzed in each group); **(E)** Immunoblot (total cell lysates) showing inhibition of proteasomal degradation by proteasomal inhibitor MG132: A498 cells were transfected with *ALK5-HA* and *VHL* followed by treatment with TGF-β, or treatment of TGF-β along with MG132 for 6 hours; **(F)** Immunoblot (total cell lysates) showing inhibition of proteasomal degradation by proteasomal inhibitor MG132: ACHN cells were transfected with *ALK5-HA*, followed by treatment with TGF-β or treatment with TGF-β along with MG132 for 6 hours; **(G)** pVHL mediates K48-linked poly-ubiquitination of ALK5: Immunoblot showing enhanced K48-linked poly-ubiquitination of ALK5 by VHL, A498 cells were transfected with indicated plasmids followed by treatment of TGF-β for 6 hours then immunoprecipitated with HA antibody and immunoblot with indicated antibody; **(H)** PLA analysis and quantification of PLA signals (K48 and HA) in A498 cells transfected with *pcDNA 3.1(+)* empty vector, *ALK5-HA* vector alone, or co-transfected with *ALK5-HA* and *VHL* vectors (mean ± SD of two experiments, 150 cells were analyzed in each group); **(I)** pVHL mediates K48-linked poly-ubiquitination of ALK5: Immunoblot showing enhanced K48-linked poly-ubiquitination of ALK5 by VHL, ACHN cells were transfected with indicated plasmids followed by treatment of TGF-β for 6 hours then immunoprecipitated with HA antibody and immune blot with indicated antibody; **(J)** PLA analysis and quantification of PLA signals (K48- and HA) in ACHN cells transfected with *pcDNA 3.1(+)* empty vector, *ALK5-HA* (mean ± SD of two experiments, 150 cells were analyzed in each group).

To explore the possibility of a functional interaction between pVHL and ALK5 proteins, A498 cells were co-transfected with *ALK5-HA* and *VHL* followed by TGF-β treatment, and ACHN cells were transfected with *ALK5-HA* followed by TGF-β treatment. Immunoprecipitation was performed in both cell lines with either *HA* or *VHL*, and the results showed that pVHL co-immunoprecipitated with ALK5 in both cases (Figure 4A, 4B, Supplementary Figure 3A and Supplementary Figure 3B).

The proximity ligation assay (PLA) was used to investigate if pVHL and ALK5 can be found to associate in a protein complex in ccRCC cells. In PLA experiments, A498 cells co-transfected with VHL and ALK5-HA showed prominent PLA signals, while control cells had no signal (Figure 4C). ACHN cells transfected with ALK5-HA showed higher PLA signals than the control cells (Figure 4D).

Collectively, these results indicated that ALK5 and pVHL are associating with each other in ccRCC cells.

### pVHL mediates proteasomal degradation of ALK5 by ubiquitination

To elucidate if proteasomal degradation of ALK5 was mediated by VHL, A498 cells were co-transfected with *VHL* and *ALK5-HA* vectors, and ACHN cells were transfected with *ALK5-HA*. After transfection, both cell lines were treated with either TGF-β or MG132 along with TGF-β. MG132, a well-known proteasomal inhibitor, inhibited proteasomal activity and increased the stability of ALK5 protein compared with the MG132 untreated cells (Figure 4E and 4F).

To validate K48-ubiquitination, an *In-vivo* ubiquitination assay was performed in A498 and ACHN cells.

A498 (VHL^-/-^) cells were subjected to immunoprecipitation after co-transfection of *ALK5-HA* and *VHL* vectors followed by TGF-β treatment. The results verified K48-linked ubiquitination of ALK5 in cells transfected with *ALK5-HA* and *VHL* when compared with cells transfected with *pcDNA* or cells transfected with *ALK5-HA* alone (Figure 4G, Supplementary Figure 4A).

In addition, *in situ* PLA experiment was performed on A498 cells (VHL^-/-^) after *ALK5-HA* overexpression. In the absence of pVHL, A498 cells showed no PLA signal (proximity of K48 and HA) in both control and *ALK5-HA* transfected cells treated with TGF-β. Further, the introduction of *VHL* along with *ALK5-HA* in A498 (VHL^-/-^) cells showed a strong PLA signal (K48 and HA) (Figure 4H).

In ACHN cells, *ALK5-HA* vector was transfected into cells, and treated with TGF-β. Following immunoprecipitation, K48-linked ubiquitination of ALK5 was also observed. Knock-down of *VHL* by siRNA reduced the K48-linked poly-ubiquitination of transiently over-expressed *ALK5-HA*, which confirmed pVHL dependent K48-linked ubiquitination of ALK5 (Figure 4I, Supplementary Figure 4B).

To support the data achieved in the ubiquitination assay, *in situ* PLA was performed in ACHN cells (VHL^+/+^) after transfecting *ALK5-HA* followed by TGF-β treatment. Cells transfected with *ALK5-HA* showed significantly higher PLA signals (proximity of K48 and HA) than the control cells (Figure 4J).

Additional experiment in ACHN cells confirmed that the VHL enhanced the ubiquitination of ALK5-FL after co-transfection of HA-tagged ubiquitin and ALK5-GST (Supplementary Figure 5).

In summary, these results indicated VHL dependent K48-linked ubiquitination of ALK5 in ccRCC cell lines in response to TGF-β.

## DISCUSSION

In the present study, we have investigated the functional role of pVHL status on TGF-β signaling in non-ccRCC as well as in ccRCC. We found that the expression of the pVHL level, in ccRCC, was significantly lower compared with non-ccRCC. The finding of similar pVHL levels in non-ccRCC and non-malignant kidney cortex tissues indicate that pVHL was not altered in non-ccRCC. This result is in line with the previous study [27]. Similar to earlier studies [28–30], the expression levels of pVHL in ccRCC (undivided), the subgroup of ccRCCs pVHL-Low, ccRCCs pVHL-High as well as in non-ccRCC, showed no association with clinicopathological factors. Although the mutation or deletion of *VHL*, is a promoting factor in the ccRCC type, *VHL* alone is not sufficient for tumor progression in RCCs [31].

Our recent study, on ccRCC, reported that increased expressions of components in canonical TGF-β signaling and in particular AlK5-ICD, a non-canonical pathway component, have a pivotal role in driving the aggressiveness of the tumors, occurrence of metastasis, and survival of the patients [25]. In the present study, we showed that the expression levels of the TGF-β signaling components, ALK5-FL and pSMAD2/3, neither were associated with clinicopathological parameters nor with the survival of non-ccRCC patients. Conversely, TGF-β signaling components were significantly involved in tumor progression and cancer-specific survival of ccRCC pVHL-Low patients. These findings strongly suggest that the status of pVHL dictates the TGF-β signaling [13, 14]. Although ccRCC pVHL-High showed clear cell histology, we observed that they deviated from the ccRCC pVHL-Low, regarding regulation of the TGF-β signaling pathway. One of the major differences between ccRCC and non-ccRCC is the levels of pVHL. It is also well known that around 50-70% of sporadic ccRCC have *VHL* gene aberration, while the remaining 30-40% of ccRCC occur with intact pVHL [32]. Apparently, ccRCC patients with an intact *VHL* gene set will mostly have unaffected pVHL. This fact is confirmed in the present study showing that a proportion of ccRCC, despite clear cell histology, has pVHL levels similar to the non-malignant kidney cortex and of non-ccRCCs that have no *VHL* deletion. This knowledge seems to be essential for choosing the treatment of RCC with targeting agents. Most of the patients with ccRCC are affected by treatment of targeting agents, while the effects of treatment on other RCCs and also non-ccRCCs are diminished [33]. Thus, the expression of TGF-β signaling components had no association to the occurrence of metastasis and survival of the patients in ccRCC pVHL-High like non-ccRCC. Altogether, our data imply that the aggressive tumor properties regulated by TGF-β signaling depend on the pVHL status in RCC. This outcome gave an indication of a possible association between expression of pVHL and expression of ALK5-FL.

Notably, in ccRCC pVHL-High, expression of PAI-1 and ALK5-ICD were found to be independent of VHL status, unlike ALK5-FL and pSMAD2/3. The expression of PAI-1 was observed to be correlated with the tumor grade and survival status of the patient. These results indicate that the PAI-1 expression is regulated by multiple pathways as previously reported [34]. Since expression of ALK5-ICD was associated with the tumor stage and survival in ccRCC pVHL-High and pVHL-Low, this indicates that occurrence of high ALK5-ICD promotes poor prognosis independent of pVHL status. These results might also indicate that ALK5-ICD is independent of pVHL status because the occurrence of the ALK5-ICD does not follow the SMAD-dependent pathway [19].

Previous studies have shown that TGF-β seems to be the target of *VHL*, but the exact mechanism was not revealed [13, 14]. The relation between pVHL status and TGF-β signaling in the clinical materials in this study was further verified by *in-vitro* cell lines studies. By overexpression versus knock-down of *VHL*, the expression of endogenous pVHL was observed to affect TGF-β signaling as well as invasive properties of ccRCC cells. We also noticed that the endogenous pVHL negatively regulated the TGF-β signaling pathway. The *in-vitro* experiment data presented in this study showed that the knocking down of *VHL* induced the downstream targets of TGF-β pathway such as PAI-1, and the presence of intact wild-type *VHL* inhibited the PAI-1 expression driven by TGF-β stimulation. Intriguingly, the presence of pVHL inversely regulated the TGF-β dependent cell invasion. *In-vitro* studies, performed in ccRCC cell lines, also showed that the pVHL interacted with ALK5, indicating that pVHL thereby might regulate the protein stability of ALK5, consequently controlling the TGF-β signaling through proteasomal degradation of ALK5 by K48-linked poly-ubiquitination. This result is supported by previous studies that pVHL can act as an E3 ubiquitination ligase, and mediates K48-linked poly-ubiquitination, and subsequent proteosomal degradation of other proteins [8, 9, 12]. Collectively, the *in-vitro* studies, performed in this report, supported data achieved in the clinical study, by showing that the invasiveness and progression of RCC were dictated by the tumor suppressor *VHL*, by revoking the TGF-β signaling pathway.

In conclusion, the novel findings presented in this study, are relevant for the understanding of the effects of systemic treatments of RCC types depending on the pVHL status. Current tyrosine kinase inhibitors have inferior effect in non-ccRCC compared with ccRCC [33, 35]. It is possible that the difference in therapy response, in part, might be associated with the status of TGF-β signaling and the expression of ALK5-ICD. Molecular analysis of individual patient’s tumors with respect to expression of ALK5-ICD and pVHL status can give additional information that might be useful for planning of the personalized treatment for patients with RCC.

## MATERIALS AND METHODS

### Patients

The study included 143 (63 male and 80 female) patients with ccRCC surgically treated between 2000 and 2009 and 58 (20 male and 38 female) patients with non-ccRCC surgically treated between 1988 and 2009. For ccRCC, the median age was 65.11 years (range 32-85 years), and the mean tumor size was 70 mm (range 12-190 mm). For non-ccRCC, the median age was 68 years (range 32-85 years) and the mean tumor size was 70 mm (range 10-190 mm) [36, 37]. Tumor size was defined as the maximum diameter determined by CT. Samples from tumor and kidney cortex tissues from the tumor-bearing kidney were obtained after nephrectomy as described previously [38]. All samples were collected after obtaining informed and authorized consent from the patients. The study was approved by the institutional review board and the ethical committee of Northern Sweden.

Tumor stage was determined following the TNM classification system 2009 [39]. In ccRCC, there were 55 patients in TNM stage I (38.46%), 24 patients in stage II (16.78%), 27 patients in stage III (18.89%), and 37 patients in stage IV (25.87%). In non-ccRCC, there were 25 (43.10%) patients in TNM stage I, 15 (25.86%) patients in stage II, 9 (15.5%) patients in stage III, and 9 (15.5%) patients in stage IV. Nuclear grade was determined according to Fuhrman et al. [40]. In ccRCC, there were 16 tumors as (11.18%) grade 1, 56 (39.16%) tumors as grade 2, 45 (31.46%) tumors as grade 3, and 26 (18.18%) tumors as grade 4. In non-ccRCC, 5 (8.62%) tumors were grade 1, 28 (48.27%) tumors as grade 2, 18 (31.03%) tumors as grade 3, and 7 (12.06%) tumors as grade 4. The RCC type was classified according to the Heidelberg consensus conference [1]. For statistical use, stage I and stage II were considered as early stage, and stage III and stage IV were considered as advanced stage. Similarly, grade I and II were considered as low grade and grade III and grade IV were considered as higher grade.

Patient follow-up performed in a scheduled program was used for survival analysis. At the last follow-up, 55 (38.40%) patients with ccRCC were alive without any indication of disease, 6 (4.2%) were alive with disease, 57 (39.9%) died of the disease, and 25 (17.5%) died due to other reasons. For non-ccRCC, 20 patients (34.5%) were alive without disease, 1 (1.7%) were alive with disease, 20 (34.5%) died of the disease, and 17 (29.30%) died due to other reasons.

### Protein extraction and analysis

Proteins from clinical samples were extracted as described previously [25]. Proteins from *in-vitro* studies were obtained by scraping the adherent cells with Radioimmunoprecipitation assay buffer (RIPA buffer), spun down at 10000 rpm for 10 minutes at 4°C, and the supernatant which consisted of proteins was collected. Proteins were analyzed using the bicinchoninic acid assay (BCA assay) (Thermo Fisher Scientific, Waltham, MA, USA) following the manufacturer’s guidelines.

### Immunoblot (IB)

Total proteins (30 μg) were separated by NuPAGE Novex 10% gels or NuPAGE Novex 12% gels (Life Technologies, Carlsbad, CA, USA) in XCell SureLock™ Mini-Cell (Life Technologies), and transferred onto nitrocellulose membrane using Transblot Turbo transfer system (Bio-Rad Laboratories, Hercules, CA, USA). Based on the antibodies, the membranes were blocked in 5% BSA or 5% non-fat milk or Odyssey blocking buffer (Licor Biosciences, Lincoln, NE, USA) diluted in Tris-Buffered Saline, for 1 hour at room temperature. The membranes were incubated overnight at 4°C with gentle agitation with the indicated primary antibodies;

TGF-βRI (or ALK5, V-22) (sc-398, Santa Cruz Biotechnology, Santa Cruz, CA, USA), which identifies ALK5-FL and ALK5-ICD, as previously reported [19], HA (CST #2367 and CST #3724, Cell Signaling Technology, Danvers, MA, USA), HA (12CA5, Roche, Basel, Switzerland), phospho-SMAD2 (CST #3108, Cell Signaling Technology), PAI-1/Serpine1 (NBP1-19773, Novus Biologicals, Littleton, CO, USA), VHL (VHL40 (*in vitro* studies), Santa Cruz Biotechnology), VHL (NB100-485 (Human clinical sample), Novus Biologicals), K48-linkage Specific (CST #4289, Cell Signaling Technology), K48-linkage Specific (ab140601, Abcam, Cambridge, United Kingdom), P4D1 Ubiquitin (CST #3936, Cell Signaling Technology) and β-actin (A5316, Sigma-Aldrich, St. Louis, MO, USA). The primary antibodies were detected using secondary antibody IRDye^®^ 800CW Goat Anti-Rabbit (Licor #926-32211, Licor Biosciences) or IRDye^®^ 680CW Goat anti-mouse (Licor #925-68070, Licor Biosciences). Odyssey CLx (Licor Biosciences) Infrared Imaging system was used to visualize the membranes, and Image Studio System™ software version 3.1 (Licor Biosciences) was utilized for densitometry. The relative numerical density values for all the proteins were calculated by dividing the density value of housekeeping protein β-actin.

### *In-vitro* cell culture studies

All cell lines utilized in this study were mycoplasma free, and used within five passages. The cells were authenticated by STR profiling (IdentiCell, Denmark). Two ccRCC cell lines, A498 (VHL^-/-^) and ACHN (VHL^+/+^), were bought from ATCC (Wesel, Germany). A498 and ACHN cells were cultured in RPMI (Sigma-Aldrich) and E-MEM (ATCC) respectively (supplemented with 10% FBS). ACHN and A498 were seeded into 10 cm plates at 1x10^6^ cells.

A498 and ACHN cells were treated with TGF-β1 (R&D system, UK) (10 ng/ml) for 1h, 2h, 3h, and 6h.

A498 and ACHN cells were transfected with C-terminally hemagglutinin (HA)-tagged ALK5 (*ALK5-HA*) or *pcDNA 3.1(+)* or *ALK5-HA* [19] and *VHL* (NM_000551.2, Origene Technologies, Inc., Rockville, MD, USA) using Lipofectamine 3000 (Thermo Fisher Scientific) following the manufacturer’s instructions. The next day, cells were starved for 12 hours, then treated with TGF-β for 6 hours, followed by protein extraction.

To knockdown *VHL*, ACHN cells were transfected with *siRNA VHL* purchased from Ambicon (Cat #:4390824, ThermoFisher Scientific) or co-transfected with *siRNA VHL* and *ALK5-HA*. *VHL siRNA* (75 μM) were transfected using RNAimax (ThermoFisher Scientific) by following manufacturer’s guidelines. After 72 hours of transfection, the cells were treated with TGF-β for 6 hours. The proteins were then collected.

For immuno-precipitation, *ALK5-HA* and *VHL* were co-transfected to A498 cell line, and *ALK5-HA* was transfected to ACHN cell line, both the cell lines were treated with TGF-β for 6 hours. Either *VHL* or HA, was immunoprecipitated from the total cell lysate using indicated antibody. Immunoblots were then probed with either HA or VHL antibody. Later, the blots were reprobed with antibody against the immunoprecipitated proteins. The primary antibodies were detected by light chain specific anti-rabbit and anti-mouse IgG antibodies conjugated with horse radish peroxidase (Jackson ImmunoResearch Laboratories, West Grove, PA, USA) and visualized by Chemiluminescence (ECL-select, Amersham Biosciences, UK).

To investigate the potential of proteasomal degradation of ALK5, A498 cells were co-transfected with *ALK5-HA* and *VHL*, and ACHN cells were transfected with *ALK5-HA*, the following day, cells were treated with TGF-β (10 ng/ml) or TGF-β along with MG132 (15 μM) (Sigma-Aldrich) for 6 hours, and cells lysates were collected.

### Proximity ligation assay (PLA)

For PLA experiment to visualize protein-protein interaction, A498 (5x10^5^) and ACHN (5x10^5^) cells were seeded onto two well chamber slide one day before transfection. A498 cells were then co-transfected with *ALK5-HA* and VHL or transfected with *pcDNA 3.1(+)* empty vector as a control, and ACHN cells were transfected with *ALK5-HA* or transfected with *pcDNA3.1(+)* empty vector as a control. In another set of experiments, A498 cells were transfected with *ALK5-HA*, or co-transfected *ALK5-HA* and *VHL* vectors, or transfected with *pcDNA 3.1(+)* empty vector as a control. ACHN cells were transfected with *ALK5-HA* or transfected with *pcDNA 3.1(+)* empty vector as a control, after 24 hours, the cells were treated with TGF-β (10 ng/ml) for 6 hours.

PLA was performed according to manufacturers' instructions (Sigma-Aldrich). In short, the cells were washed with PBS, fixed using 4% formaldehyde and permeabilized using Triton-X. The cells were blocked with 5% BSA in 0.2% Triton-X. After blocking, the cells were incubated with HA antibody (CST #3724, Cell Signaling Technology) and VHL G7 antibody (SC-17780, Santa Cruz Biotechnology) or HA antibody (CST #2367, Cell Signaling Technology) and K48 (ab140601, Abcam) for 24 hours in a humidity chamber at 4°C. The cells were washed with PBS and incubated with rabbit plus and mouse minus probes. Detection of PLA signal was performed using the kit purchased from Sigma-Aldrich. Slides were scanned using a confocal microscope Zeiss 710 Meta (Carl Zeiss MicroImaging, Inc.) and zen-2010 software. The Images were acquired at 40X and 63X. The images were analyzed and represented graphically using Duolink Image Tool (Sigma-Aldrich).

### Invasion assay

Invasion assay kit was purchased from Cell Biolabs (CBA-110-COL, San Diego, CA, USA), and the assay was performed according to the manufacturer's protocol. In short, 48 hours after transfection, cells were starved, trypsinized and added to the upper chamber of invasion assay containing media with 2% FBS, and the wells were filled with media with 10% FBS, the cells were stimulated with TGF-β (10 ng/ml), unstimulated cells served as controls. The cells were stained and eluted, and the resulting solution’s optical density (OD) was measured at 560 nm. Cells were visualized under a Leitz light microscope (Germany) at magnification 10X. Images were captured using DpxViewPro software (Denmark).

### *In-vivo* ubiquitination assay

A498 and ACHN Cells (5x10^6^) were seeded one day before transfection. A498 cells were transfected with *pcDNA 3.1(+)* or co-transfected with *ALK5-HA* and *VHL* or transfected with *ALK5-HA* alone. ACHN cells were transfected with *pcDNA 3.1(+)* or co-transfected with siRNA control and *ALK5-HA* or transfected with *ALK5-HA* or co-transfected with *siVHL* and *ALK5-HA* using Lipofectamine 3000 (Thermo Fisher Scientific) following the manufacturer’s instructions. After 24 hours following transfection, cells were starved, and treated with TGF-β for 6 hours, and then cell pellets were collected. In brief, cell pellets were boiled at 95°C in 200 μl of PBS containing 1% SDS for 10 minutes. After boiling, 1800 μl PBS with 0.5% NP-40 along with A-protein (1:100) and Pefabloc (1:200) was added. The samples were then centrifuged at 13000 rpm for 10 minutes at 4°C. Then the supernatants were subjected to immunoprecipitation, followed by immunoblotting. Ubiquitination assay was performed as previously described [41].

### Statistical analysis

For clinical studies, statistical analysis was performed utilizing IBM SPSS Statistics 24.0. For the cell culture experiments the Student’s t-test was used. The difference in expression of two independent variables was analyzed by the Mann-Whitney U-test. Kaplan-Meier curves were used to express survival times, and the log-rank test was used to compare the survival times. For all the tests, a *P* value less than 0.05 was considered significant.

## SUPPLEMENTARY MATERIALS FIGURES


