# Supplementary material for: VHL status regulates transforming growth factor-β signaling pathways in renal cell carcinoma
Source: Oncotarget. 2018 Mar 27;9(23):16297–310. doi: 10.18632/oncotarget.24631 (PMC5893241; doi:10.18632/oncotarget.24631)
Supplement: Supplementary file 1 [file oncotarget-09-16297-s001.pdf]

VHL status regulates transforming growth factor-β signaling pathways in renal cell carcinoma

SUPPLEMENTARY MATERIALS

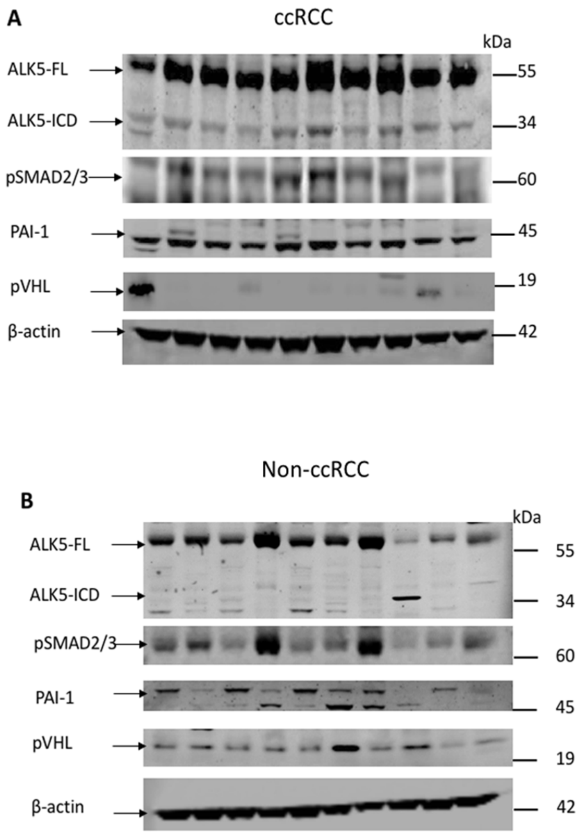

Supplementary Figure 1: (A) Representative immunoblots ccRCC tumors (10 out of 154 loaded in lane 1-10) showing expression of ALK5-FL, ALK5-ICD, pSMAD2/3, and PAI-1 in ccRCC tissues. β-actin served as internal loading control; **(B)** Non-ccRCC tumor samples (10 out of 54 loaded in lane 1-10) showing expression of ALK5-FL, ALK5-ICD, pSMAD2/3, and PAI-1 in ccRCC tissues. β-actin served as internal loading control.

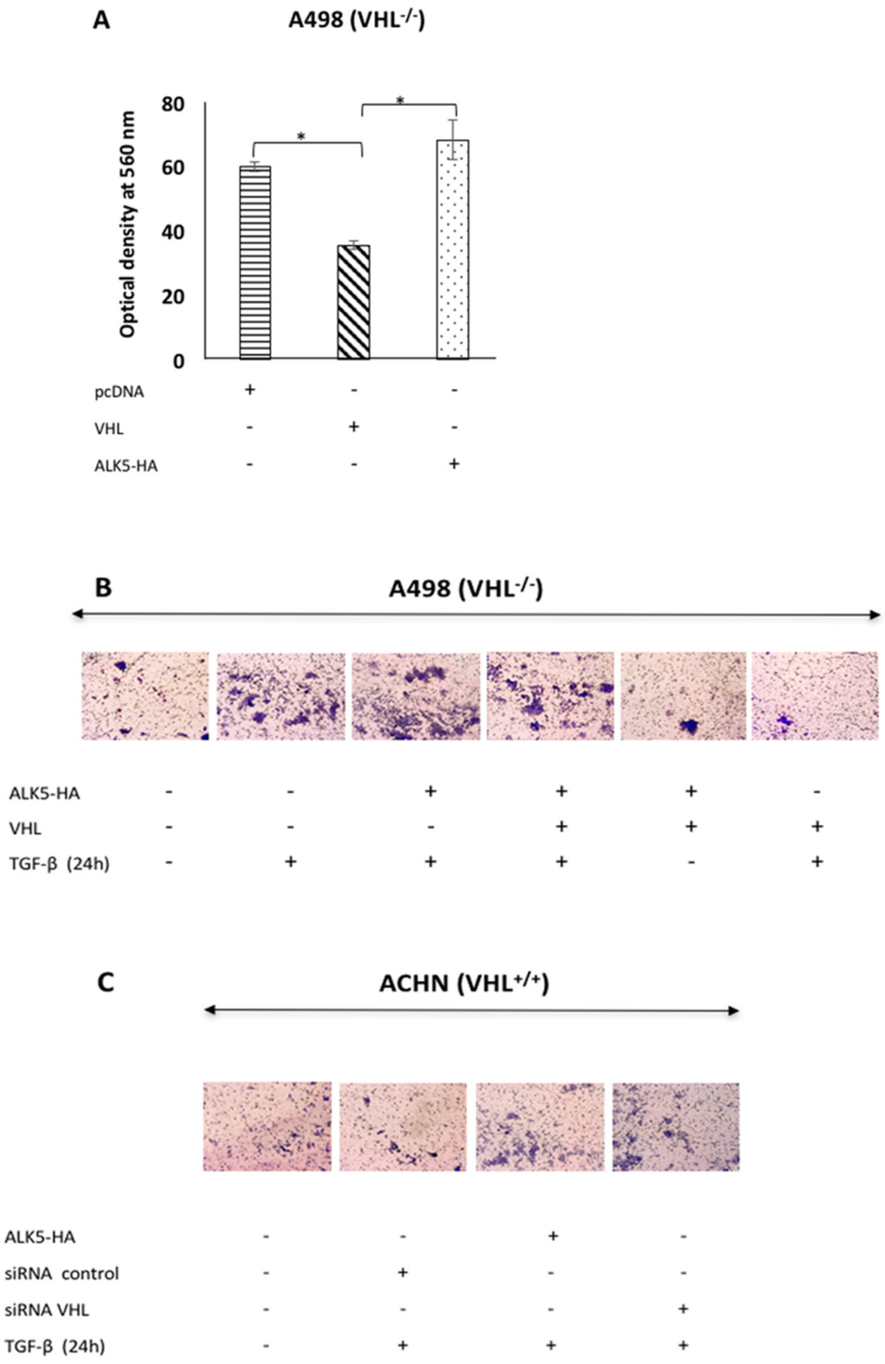

Supplementary Figure 2: (A) Invasion assay (for comparison with Figure 2E) showing the invasiveness in cells transfected with *pcDNA 3. 1(+)* empty vector or *VHL* or *ALK5-HA* without treatment of TGF-β in A498 cells (n=2 independent experiments *P< 0.05); **(B)** Representative picture of invasion assay showing the invasiveness of A498 cells treated with indicated plasmids along with TGF-β; **(C)** Representative picture of invasion assay showing the invasiveness of ACHN cells treated with indicated plasmids along with TGF-β.

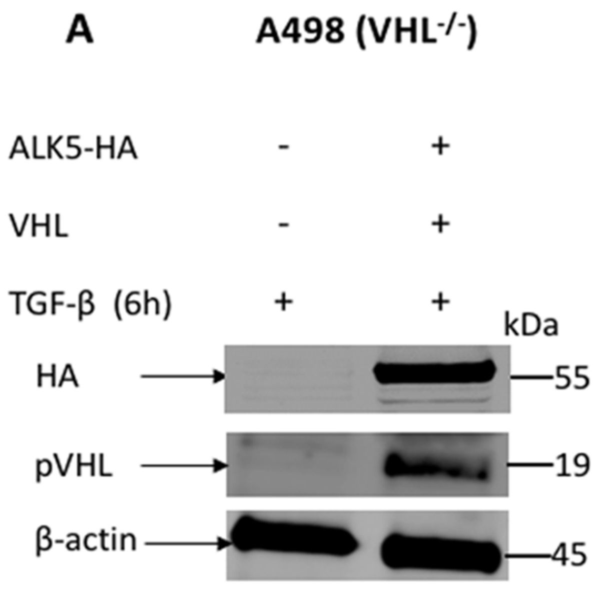

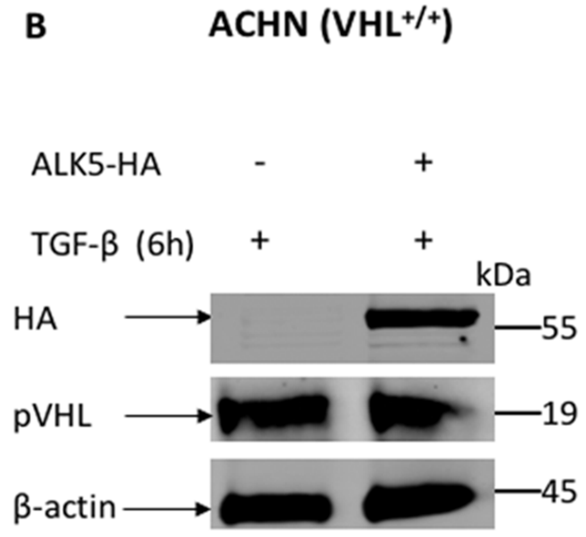

Supplementary Figure 3: (A) Input immunoblots (total cell lysates) for IP showing protein expression of HA, pVHL, and β-actin in A498 cells after transfection of indicated vectors, and with or without treatment TGF-β for 6 hours; **(B)** Input immunoblots (total cell lysates) for IP showing protein expression of HA, pVHL, and β-actin in ACHN cells after transfection of indicated vectors, and with or without treatment TGF-β for 6 hours.

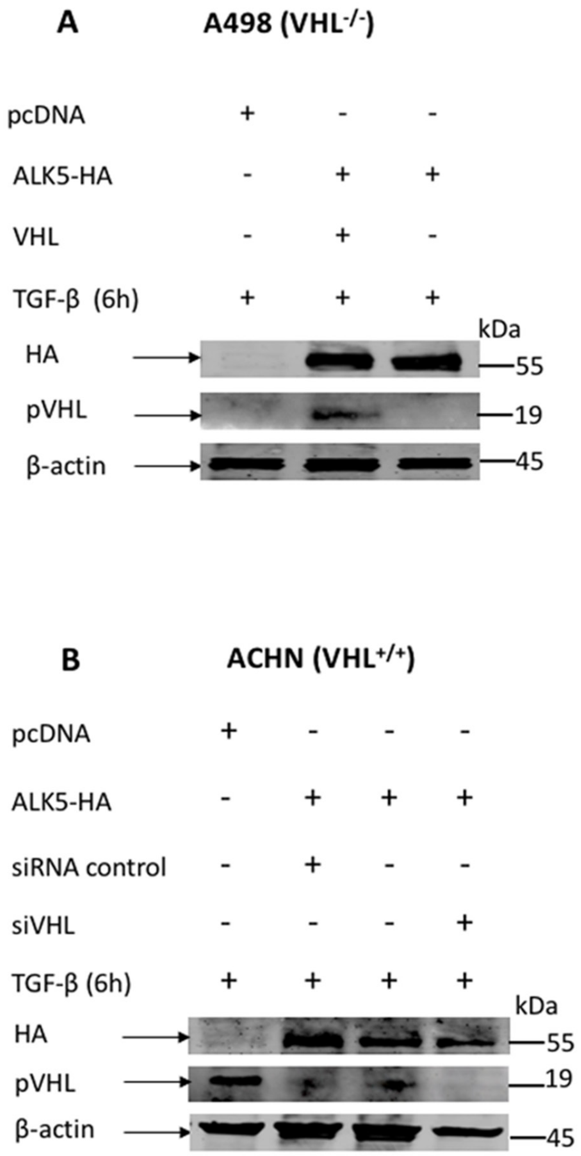

Supplementary Figure 4: **(A)** Input immunoblots for ubiquitination assay showing protein expression of HA, pVHL, and β-actin in A498 cells after transfection of indicated vectors with or without treatment TGF-β for 6 hours; **(B)** Input immunoblots for ubiquitination assay showing protein expression of HA, pVHL, and β-actin in ACHN cells after transfection of indicated vectors with or without treatment TGF-β for 6 hours.

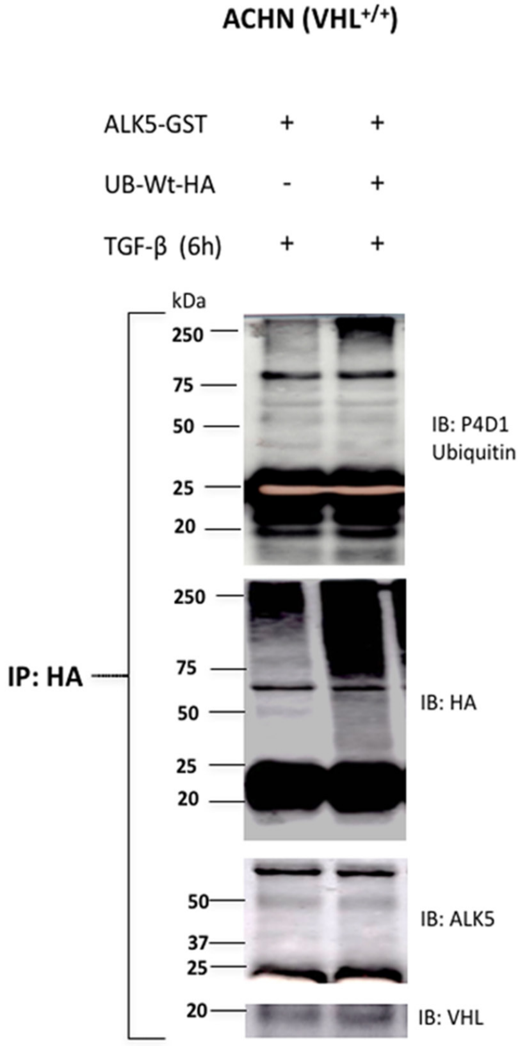

Supplementary Figure 5: pVHL mediates K48-linked poly-ubiquitination of ALK5: immunoblot showing enhanced K48-linked poly-ubiquitination of ALK5 by pVHL; ACHN cells were transfected with indicated vectors, followed by treatment with TGF-β for 6 hours. Then total cell lysates were subjected to immunoprecipitated with HA antibody and immunoblot with indicated antibody.
